# Primary and acquired resistance to EGFR-targeted therapies in colorectal cancer: impact on future treatment strategies

**DOI:** 10.1007/s00109-014-1161-2

**Published:** 2014-05-10

**Authors:** Simonetta M. Leto, Livio Trusolino

**Affiliations:** 1 Department of Oncology, University of Torino Medical School, 10060 Candiolo, Torino Italy; 2 Laboratory of Molecular Pharmacology, Candiolo Cancer Institute—FPO IRCCS, Strada Provinciale 142, km 3.95, 10060 Candiolo, Torino Italy

**Keywords:** Colorectal cancer, Targeted therapy, Anti-EGFR antibodies, Primary resistance, Secondary resistance, Response biomarkers

## Abstract

Only approximately 10 % of genetically unselected patients with chemorefractory metastatic colorectal cancer experience tumor regression when treated with the anti-epidermal growth factor receptor (EGFR) antibodies cetuximab or panitumumab (“primary” or “de novo” resistance). Moreover, nearly all patients whose tumors initially respond inevitably become refractory (“secondary” or “acquired” resistance). An ever-increasing number of predictors of both primary and acquired resistance to anti-EGFR antibodies have been described, and it is now evident that most of the underlying mechanisms significantly overlap. By trying to extrapolate a unifying perspective out of many idiosyncratic details, here, we discuss the molecular underpinnings of therapeutic resistance, summarize research efforts aimed to improve patient selection, and present alternative therapeutic strategies that are now under development to increase response and combat relapse.

## Introduction

Colorectal cancer is the second commonest cancer worldwide, and the metastatic disease accounts for up to 20 % of newly diagnosed patients or further develops in 50 % of cases, with a median overall survival (OS) of approximately 20 months [1-5].

The clinical outcome of patients with metastatic colorectal cancer (mCRC) has been improved by the introduction of cetuximab and panitumumab, two monoclonal antibodies (moAbs) targeting the epidermal growth factor receptor (EGFR/ErbB1/HER1), given in combination with chemotherapy or, when other options are exhausted, as monotherapy [6-8].

**Fig. 1:**
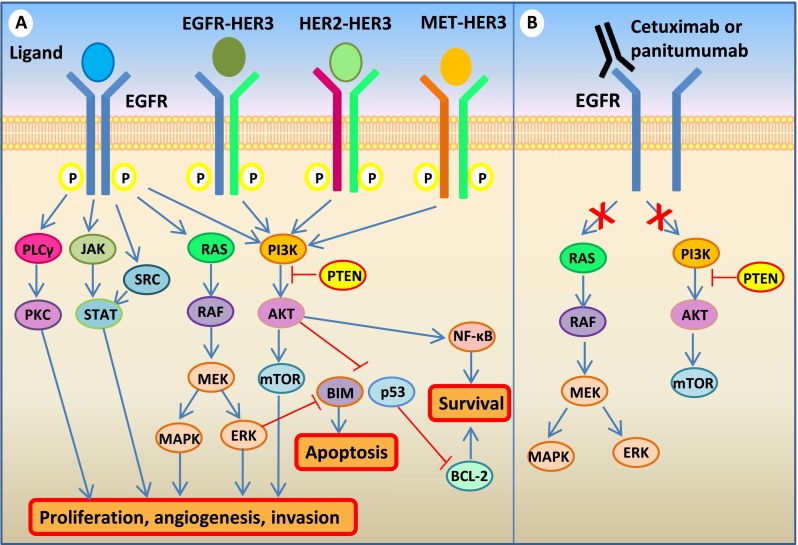
EGFR signaling pathways. **a** Upon ligand binding and consequent homo- and hetero-dimerization, ErbB family members can activate a number of pathways, including the RAS-RAF-MEK-ERK and the PI3K-AKT-mTOR axes, the SRC family kinases (SFKs), PLCγ-PKC, and STATs, driving cell proliferation and/or influencing apoptosis. **b** By binding the extracellular domain of EGFR, both cetuximab and panitumumab prevent ligand-induced activation of downstream signaling

EGFR is a member of the ErbB family of receptor tyrosine kinases (RTKs), which also includes HER2/neu (ERBB2), HER3 (ErbB3), and HER4 (ErbB4) [9]. EGF or other EGF-like ligands trigger homo- and hetero-dimerization of EGFR with other ErbB members, which activates a mitogenic and antiapoptotic signaling cascade via several pathways, including not only the RAS-RAF-MEK-ERK and the PI3K-AKT-mTOR axes but also SRC family kinases, PLCγ-PKC, and STATs [9, 10]. Such activation stimulates key processes involved in tumor growth and progression, including proliferation, angiogenesis, invasion, and metastasis [11] (Fig. 1).

When used as monotherapy in genetically unselected patients with chemotherapy-refractory mCRC, cetuximab and panitumumab achieve clinically meaningful response rates (RRs) of approximately 10 % [7, 8, 12]. Unlike other tumor types such as non-small cell lung cancers (NSCLCs) or melanomas, in which target mutations are associated with massive regressions following treatment with specific inhibitors [13, 14], genetic alterations of EGFR are extremely infrequent in colorectal tumors.

The complex and thin boundary between primary and acquired resistance is determined by the evidence of an initial response to treatment. If refractoriness to therapy is present at baseline, this is defined as primary (also known as de novo) resistance and can be explained by resistance-conferring factors preexisting in the bulk of tumor cells. Acquired (or secondary) resistance refers to disease progression in the face of ongoing treatment that was initially effective and can be caused by mutations arising during treatment as well as through other various adaptive nongenetic responses [15, 16]. In the case of colorectal cancer, acquired resistance typically occurs within 3–18 months after treatment initiation [7, 8].

Starting with seminal observations in 2006–2007 [17, 18], a large body of evidence has described different biomarkers of primary resistance to anti-EGFR moAbs in mCRC patients, leading to exclusion from treatment of a number of molecularly defined nonresponders [19, 20]. The field of acquired resistance has received preclinical and clinical attention much more recently, with the emergence of new insights only in the last 2 years.

In this review, we will appraise the current knowledge on primary and acquired resistance to anti-EGFR moAbs in mCRC, from initial mechanistic exploration to clinical applications, and will highlight emerging lines of investigation aimed at improving response and delay relapse in this tumor setting.

## Molecular mechanisms of resistance to anti-EGFR antibodies in patients with metastatic colorectal cancer

**Fig. 2:**
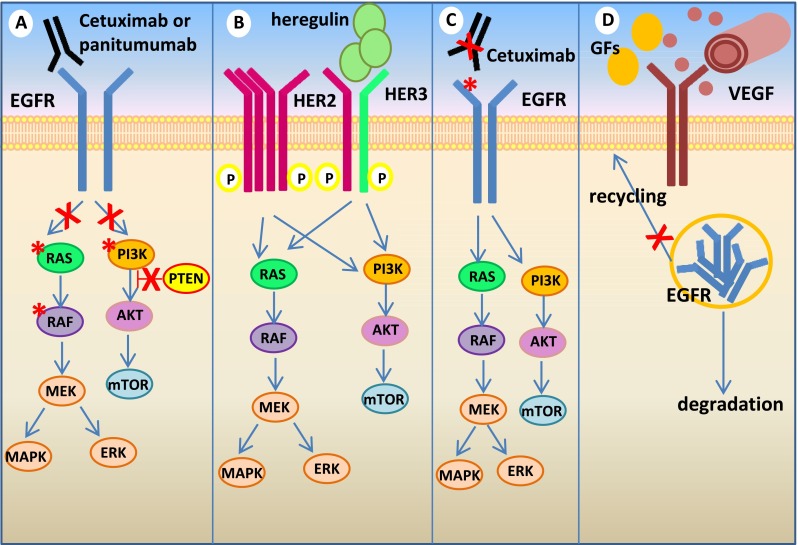
Mechanisms of resistance to anti-EGFR moAbs in mCRC. **a** Activating mutations of EGFR effectors, such as KRAS (by either point mutations or gene amplification), BRAF and PI3KCA, or PTEN loss of function, cause persistent activation of downstream signaling despite EGFR inhibition. **b** Aberrant activation (by either receptor gene amplification or high ligand levels) of alternative receptors, such as HER2 or MET (not shown), can bypass EGFR inhibition and mediate downstream pathway activation. **c** Additional genetic alterations within the target oncogene may abrogate drug binding. The EGFR S492R mutation inhibits cetuximab but not panitumumab binding, mediating acquired resistance to the former but not the latter in mCRC patients. **d** Other mechanisms of resistance may be “pathway independent,” such as altered angiogenesis (through increased secretion of VEGF or activation of VEGFR-1/2), dysregulation of EGFR recycling (with consequent increase of EGFR degradation), or tumor-stroma interactions (i.e., through increased release of antiapoptotic growth factors and cytokines, such as HGF)

In general terms, the commonest mechanisms of resistance to inhibition of receptor tyrosine kinases (RTKs) involve genomic alterations affecting downstream effectors, such as *KRAS* and *PIK3CA* mutations, with consequent constitutive pathway hyperactivation. Notably, the KRAS and PI3K signaling cascades can also be activated by upstream RTKs other than EGFR [21], leading to an oncogenic shift [22]. In both cases, the primary drug target remains unaltered and continues to be inhibited while an alternative signal transducer becomes activated, bypassing the consequences of EGFR inhibition [16, 23] (Fig. 2a, b).

Importantly, it is increasingly recognized that tumors can contain a high degree of genetic and molecular heterogeneity within the same lesion [24]. Thus, secondary resistance can arise not only through acquisition of de novo genetic lesions over the course of therapy but also through treatment-induced selection of resistant minor subpopulations of cells that are intrinsically insensitive and already present in the original tumor [25]. If secondary resistance may be nothing but the emergence, under drug pressure, of rare tumor subsets featuring primary resistance, then most of the molecular mechanisms of primary and acquired resistance should overlap. Accordingly, hereinafter, we provide a description of resistance predictors as a whole, specifying for each biomarker when it has been reported in both cases. We will also focus on current research efforts aimed at developing alternative strategies to circumvent such resistances in patients with no other therapeutic options. Table 1 summarizes the main biomarkers of primary and acquired resistance observed in mCRC patients and describes potential alternative strategies proposed by different approaches.

**Table 1:** Biomarkers of primary and acquired resistance to anti-EGFR moAbs in mCRC patients and potential alternative therapeutic strategies

Biomarker	Scientific approach	Alternative strategies proposed	References
Primary resistance			
* KRAS* mutations	*KRAS* mutant cell lines in vitro and in vivo	Combination of EGFR and MEK inhibitors was more effective than either agent alone in reducing cell viability in vitro.	[18]
		Combination of dasatinib (SFK inhibitor) with cetuximab induced decreased proliferation and enhanced apoptosis in vitro, tumor growth delay but not regression in vivo.	[51]
	Synthetic lethal interactions in *KRAS* mutant cell lines	Mutant *KRAS* cells exhibited selective sensitivity to suppression of the mitocondrial apoptosis-regulator STK33. Studies to develop STK33 inhibitors are required.	[45]
		*RAS*- mutant cells were sensitive to proteasome and mitotic perturbations. PLK1 inhibition attenuated tumor growth in vivo.	[46]
		Combined IGF-IR and MEK inhibition induced partial tumor regression in vivo.	[49]
		TAK1 inhibition promoted apoptosis in KRAS-dependent APC-mutant CRC cells and tumor regression in vivo.	[48]
		Proteasome and topoisomerase inhibitors selectively impaired cell viability (GATA2 and CDC6 could be potential new targets).	[44]
		Combined BCL-XL and MEK inhibition promoted tumor regression in vivo.	[47]
	Patient-derived xenografts of *RAS* mutant CRCs	Inhibition of MEK and PI3K/mTOR induced tumor growth delay but not regression. This strategy may retard progression in patients.	[43]
BRAF mutations	*KRAS* or *BRAF* mutant cells, mouse xenografts and GEMMs.	Combined targeting of BCL-2/BCL-XL and TORC1/2 induced selective apoptosis in vitro and tumor regression in vivo.	[50]
	*BRAF* V600E CRC models	Combined BRAF and EGFR inhibition was synergistic in vitro and in vivo.	[52, 58, 59]
		Calfizomib (proteasome inhibitor) reduced cell viability in vitro and suppressed tumor growth in vivo.	[64]
	Cell lines with concurrent *PIK3CA* mutations or PTEN loss/BRAF V600E GEMMs	Combination therapy with BRAF and PI3K inhibitors induced apoptosis in vitro, delayed tumor growth in vivo and caused tumor regression in GEMMs.	[60, 62, 63]
PIK3CA mutations or PTEN loss	Cells carrying *PIK3CA* mutations or PTEN loss but not BRAF/KRAS mutations	Adjuvant low-dose aspirin in PIK3CA-mutant patients improved survival. Further prospective studies are required.	[85, 86]
HER2 amplification	*HER2*-amplified patient-derived xenografts	Combination of cetuximab/pertuzumab with lapatinib induced overt long-lasting tumor regression.	[91]
MET activation	HGF-overexpressing cells	Co-treatment with cetuximab and MET inhibitors induced marked tumor regression of HGF-overexpressing cells in vivo.	[105]
	*MET* amplified patient-derived xenografts	MET inhibition achieved long-lasting abolition of tumor growth in vivo.	[104]
Acquired resistance			
* EGFR* mutations	Mutations in the EC domain (S492R) and in the kinase domain (codons 714 and 794) of EGFR found in patients	Panitumumab remained active in a patient with S492R mutation, which abrogated cetuximab binding.	[41, 109]
* RAS/BRAF* activation	CRC cell lines with acquired *KRAS/BRAF* point mutations and/or *KRAS* amplification and one patient-derived xenograft	Combination of cetuximab with pimasertib (MEK inhibitor) induced moderate tumor shrinkage in vivo.	[40]
* HER2* activation	Cells with high heregulin levels or *HER2* amplification	Pertuzumab/lapatinib restored sensitivity to cetuximab in vitro.	[92]
* MET* activation	*MET* amplified patient-derived xenografts	Combined inhibition of MET and EGFR induced long-lasting disease stabilization in vivo	[104]

### RAS

The RAS family includes three small GTPases (KRAS, NRAS, and HRAS) responsible for coupling EGFR to the RAF/MEK/ERK pathway [22]. Several retrospective analyses have described *KRAS* mutations in exon 2 (codons 12 and 13), which are found in approximately 40–45 % of CRCs [20, 26], as major determinants of primary resistance to cetuximab or panitumumab [17, 27-29]. The robust predictive power of such correlations, despite being obtained in retrospective studies, was sufficient to convince both the US Food and Drug Administration and the European Medicines Agency to approve the use of anti-EGFR moAbs only in the subset of *KRAS* wild-type colorectal cancers [26, 30-34].

Although exclusion of patients with *KRAS* (exon 2)-mutant tumors has arithmetically increased the percentage of responders up to 13–17 %, most *KRAS* wild-type tumors still do not respond to anti-EGFR moAbs [26, 32]. Additional rare mutations of *KRAS*, as well as mutations of *NRAS*, have been associated with primary resistance to treatment. The relatively high cumulative frequency of rare *KRAS* mutations and *NRAS* mutations, coupled with initial successful validation in prospective trials, strongly advocates prompt incorporation of such biomarkers into clinical practice as negative predictors [35]. A very low frequency of *KRAS* amplification (0.7 %) has also been reported and found to correlate with primary resistance [36].

*KRAS* point mutations and gene copy number gains are responsible not only for primary but also for acquired resistance in 38–60 % of patients who relapse on cetuximab or panitumumab [37-39]. Intriguingly, such mutations presumably are either present in a clonal subpopulation within the tumor before treatment initiation [37, 38] or raise as a consequence of continued mutagenesis over the course of therapy [38, 39]. *KRAS* alterations could be identified noninvasively 5–10 months before radiographic disease progression by analyzing cell-free circulating tumor DNA (ctDNA) [37, 38]. Using this approach, two recent studies have highlighted the emergence of several independent clones carrying heterogeneous patterns of *KRAS* and *NRAS* mutations concomitantly associated with acquired resistance to EGFR blockade [40, 41].

Currently, *KRAS*-mutant patients are treated with chemotherapy (with or without antiangiogenic therapy using the anti-VEGF moAb bevacizumab), but if intensive regimens are not tolerated or relapse occurs, the remaining treatment option is best supportive care [42]. To date, direct inhibitors of mutant KRAS protein are not yet available; therefore, multiple efforts have been made at the preclinical level by approaches as different as targeting downstream effectors such as MEK and PI3K [43], exploiting synthetic lethal interactions [44-49] or using high-throughput drug screens [50]. Of note, most of these studies showed that simultaneous targeting of two different pathways induced some responses in *KRAS* mutant CRC mouse models, albeit rarely with overt tumor regressions [51] (see Table 1); most of these approaches are currently under evaluation in phase I/II clinical trials (NCT01085331, http://clinicaltrials.gov/ct2/show/NCT01085331?term=NCT01085331&rank=1; NCT01390818, http://clinicaltrials.gov/ct2/results?term=NCT01390818&Search=Search; NCT02039336, http://clinicaltrials.gov/ct2/show/NCT02039336?term=NCT02039336&rank=1). In the case of secondary resistance due to *RAS* mutations, preclinical evidence suggests that early initiation of a combinational targeting of EGFR and MEK could delay or reverse the emergence of resistance [40].

### BRAF

Mutations of *BRAF*, which encodes the cytoplasmic serine/threonine kinase immediately downstream of RAS, are found in 4–13 % of advanced CRCs and are usually mutually exclusive with *KRAS* mutations [20, 52].

The *BRAF* V600E mutation has been described as a predictor of tumor aggressiveness in metastatic disease [33, 52, 53] and also of low RRs to cetuximab and panitumumab [18, 20, 52, 53]. However, the predictive impact of *BRAF* mutations is tempered by their low prevalence and is further biased by the prominent role of mutant *BRAF* as a negative prognostic biomarker [54]. Overall, the predictive power of this alteration remains immature and requires further prospective endorsement before clinical applicability [20, 33, 52, 55].

Although, unlike RAS, BRAF can be efficiently blocked by clinically approved small-molecule inhibitors, no targeted therapeutic options are currently available for *BRAF* mutant CRC. In contrast to dramatic responses obtained in *BRAF* V600E-mutant melanomas (RR of 48 to 67 %) [13, 56], selective BRAF inhibitors such as vemurafenib have failed in *BRAF*-mutant CRCs (RR of 5 %) [57]; this lack of efficacy has been ascribed to the feedback activation of EGFR, which ensues as a consequence of BRAF inactivation and leads to EGFR-dependent compensatory signals [58, 59]. Accordingly, preclinical studies have provided a proof of principle that the combined inhibition of EGFR and BRAF can be synergistic in *BRAF*-mutant CRCs; however, it is worth noting that the best responses of CRC cell xenografts to such combinations were only disease stabilizations or mild tumor regressions [52, 58-60]. At the clinical level, a recent case report sustained the rationale of combined therapy with vemurafenib and cetuximab in *BRAF* V600E-mutant mCRC patients [61], and a pilot study of vemurafenib and panitumumab in this disease setting is currently recruiting participants (NCT01791309, http://clinicaltrials.gov/ct2/results?term=NCT01791309&Search=Search). From the diagnostic viewpoint, the feedback activation of EGFR upon BRAF inhibition likely implies that EGFR expression and phosphorylation levels may be potential predictors of response to vemurafenib monotherapy in *BRAF*-mutant mCRC patients [58, 59]. Other combinatorial approaches needing further testing or already under clinical evaluation [50, 62-64] are listed in Table 1.

*BRAF* mutations could be also detected noninvasively by ctDNA analysis, together with concomitant *KRAS* and *NRAS* mutations [40, 41], in patients who objectively responded to anti-EGFR therapy but subsequently relapsed. This indicates that the emergence of *BRAF*-mutant-resistant subclones also sustains secondary resistance.

### PI3K-AKT-PTEN pathway

PI3Ks are a family of lipid kinases; in particular, class IA PI3Ks can be activated by different RTKs [65], but also through RAS association [66] or signaling from G protein-coupled receptors [9].

Class IA PI3Ks consist of heterodimeric proteins composed of a regulatory (p85) and a catalytic (p110) subunit [67]. Activating mutations of *PIK3CA* (encoding p110α) have been found in 10–20 % of CRCs [20, 68-70]; most of them occur in exons 9 and 20, respectively, in the helical and kinase domain [68, 71]. Sartore-Bianchi and colleagues performed a retrospective analysis of 110 mCRC patients treated with cetuximab or panitumumab, reporting a statistically significant association between *PIK3CA* mutations and primary resistance to treatment within *KRAS* wild-type tumors. In this study, 11 out of 15 mutations were found in exon 20 (73.3 %) and only 4 in exon 9 (26.7 %) [72]. Another study, in which a majority of exon 9 mutations was reported, did not confirm such correlation [70]. These conflicting reports were then reconciled by a large retrospective consortium analysis on 1,022 tumor samples which showed that, in the *KRAS* wild-type subpopulation, only *PIK3CA* exon 20 mutations may be predictive of lack response to cetuximab (RR of 0 % in mutant vs 36.8 % in wild-type cases) [20]. This study also described a strong association between *PIK3CA* exon 9 (but not exon 20) mutations and *KRAS* mutations, suggesting the lack of independent influence of *PIK3CA* exon 9 mutations on cetuximab efficacy.

Loss of function of PTEN, which antagonizes PI3K activity, occurs in 30 % of sporadic CRCs through a variety of mechanisms [73, 74]. PTEN inactivation (usually assessed as lack of protein expression) has been associated with nonresponsiveness to anti-EGFR moAbs in mCRC patients in several studies [19, 73, 75, 76], whereas others have only reported a prognostic role [53]. In summary, both *PIK3CA* exon 20 mutations and loss of PTEN expression are promising predictors of tumor suitability for anti-EGFR therapies. However, due to the low incidence of exon 20 mutations (2–5 %) [77] and lack of a consensus method for PTEN expression analysis [20, 73, 76, 78, 79], further prospective trials are required to challenge the clinical utility of PI3K pathway activation as a negative response predictor.

In principle, patients harboring *PIK3CA* mutations or PTEN loss of function, without concomitant *KRAS/BRAF* mutations, may benefit from targeted treatments against PI3K or PI3K-downstream effectors such as mTOR or AKT [80]; however, emerging clinical data have shown only minimal single-agent activity of such inhibitors at tolerated doses [81-83]. It is likely that mTOR kinase, AKT, pan-PI3K, or isoform-specific PI3K inhibitors will provide greater therapeutic index when combined with RTK inhibitors [84]. Phase I/II studies testing mTOR inhibitors, such as everolimus or temsirolimus, in combination with RTK inhibitors or anti-EGF moAbs plus chemotherapy in mCRC patients are underway (NCT01154335, http://clinicaltrials.gov/ct2/show/NCT01154335?term=colorectal+cancer&rank=33; NCT01139138, http://clinicaltrials.gov/ct2/show/NCT01139138?term=colorectal+cancer&rank=67; NCT01387880, http://clinicaltrials.gov/ct2/show/NCT01387880?term=everolimus+AND+colorectal+cancer&rank=2; NCT00827684, http://clinicaltrials.gov/ct2/show/NCT00827684?term=everolimus+AND+colorectal+cancer&rank=9).

Finally, recent observational studies have shown that adjuvant low-dose aspirin improved survival in patients with *PIK3CA*-mutant tumors [85-87]; this sensitivity requires further prospective evaluation and could be at least partially explained by the fact that PI3K-AKT seems to induce NF-ĸB-dependent transcriptional upregulation of COX2, which has been demonstrated to display pro-survival activity in CRC cells [87-89]. Therefore, a *PIK3CA*-mutant context may render CRC cells susceptible to apoptosis by aspirin-mediated COX2 inhibition.

To our knowledge, no alterations in the PI3K/AKT pathway have been associated with acquired resistance thus far.

### HER2

It has been calculated that, among nonresponsive patients, 70 % bear tumors harboring at least one genetic alteration in the four abovementioned markers: *KRAS*, *NRAS*, *BRAF*, and *PIK3CA* [19]; therefore, the remaining 30 % of “quadruple negative” resistant cases display still-unidentified features that sustain lack of response.

HER2 is the only member of the ErbB family that does not bind ligands; it is activated via hetero-dimerization with the other ligand-bound receptors [10], with the strongest mitogenic signals created by HER2-HER3 heterodimers; HER2 overexpression, usually caused by gene amplification, allows HER2 activation even in the absence of ligand bound to the other partners [90].

Two independent studies have recently shown that *HER2* amplification is a predictor of poor sensitivity to anti-EGFR antibodies [91, 92]. By performing genotype-response correlations in a preclinical platform of patient-derived metastatic CRC xenografts (xenopatients), Bertotti and colleagues identified *HER2* amplification as a biomarker of resistance to cetuximab within a quadruple negative population. Concomitantly, using a combination of resistant clones from cetuximab-sensitive cell lines and plasma and tissue samples from cetuximab-treated mCRC patients, Yonesaka and colleagues reported aberrant HER2 signaling (by either *HER2* amplification or through overproduction of the HER3-activating ligand heregulin) as a mediator of lack of response [92]. In retrospective studies, patients with tumors featuring *HER2* amplification or heregulin overexpression and treated with cetuximab or panitumumab experienced disease progression and shorter progression-free and overall survival compared with *HER2* wild-type cases [91-93].

Interestingly, in patients with acquired resistance, *HER2* amplification was present in a small percentage of pretreatment tumor cells (14 %) that considerably increased in posttreatment samples (71 %). Similarly, heregulin levels, evaluated both in plasma and tumor specimens, were found to be significantly higher in patients that relapsed on anti-EGFR therapy [92]. This indicates that enhanced HER2 signaling confers both primary and acquired resistance.

Active HER2 also contributes to unleashing the oncogenic properties of *HER3* mutations, which have been recently identified in about 11 % of colon cancers [94]. A “dosage effect” may be envisioned whereby low-grade *HER2* amplification or low levels of heregulin, which alone would be insufficient to sustain therapeutic resistance, might in fact decrease responsiveness to EGFR inhibition by collaborating with coexisting *HER3* mutations. Anti-HER3 antibodies and small molecules are now available and have been shown to effectively impair HER3-mediated signals and tumor progression in preclinical studies in vivo [94]. Therefore, *HER3* mutations in CRC deserve further exploration as new potential biomarkers of resistance to EGFR targeted therapies as well as new predictors of response to alternative treatment options.

Therapeutically, only the dual targeting of HER2 and EGFR by combining a small-molecule inhibitor, such as the dual EGFR/HER2 inhibitor lapatinib, with a moAb, such as cetuximab or pertuzumab, induced overt and long-lasting tumor regressions in proof-of-concept trials in *HER2*-amplified xenopatients [91]. This finding led to the design and execution of a clinical trial that is currently assessing the activity and efficacy of a trastuzumab-lapatinib combination in mCRC patients with *KRAS* wild-type, *HER2*-amplified, cetuximab-resistant tumors (https://www.clinicaltrialsregister.eu/ctr-search/trial/2012-002128-33/IT). A similar study, in which a combination of trastuzumab and irinotecan was tested in patients with HER2-overexpressing advanced colorectal cancers, has been recently completed (NCT00003995, http://clinicaltrials.gov/ct2/results?term=NCT00003995&Search=Search). It is likely that also heregulin-driven tumors lacking *HER2* amplification may benefit from HER2-directed therapies [92, 95, 96], although the definition of proper cutoff levels for ligand expression will be necessary before starting further clinical studies.

### MET

The RTK MET and its ligand, hepatocyte growth factor (HGF), can activate a number of pathways, including the RAS-BRAF-ERK cascade, the PI3K-AKT axis, SRC, and STAT signaling [97]; these signaling networks collectively influence multiple key processes in cancer such as proliferation, apoptosis, invasion, and angiogenesis [98, 99]. Aberrant activation of MET may occur by several mechanisms, including *MET* amplification and/or increased HGF expression/activity [97], and has been widely described as a cause of both primary and acquired resistance to EGFR inhibitors in NSCLCs carrying *EGFR* mutations [100-102].

HGF-induced MET activation as a mechanism of attenuated sensitivity to cetuximab in CRC has been reported by preclinical studies using either CRC cell lines [103, 104] or, more recently, CRC spheroids enriched in cancer stem cells [105]. In these studies, only the simultaneous blockade of both MET and EGFR effectively impaired tumor growth in vivo. Based on publicly available gene expression data, cetuximab resistance mediated by HGF overexpression may be also relevant in mCRC patients [105]. However, similar to that discussed for heregulin, the investigation of such candidate biomarker requires the definition of methods and the categorization of expression cutoffs before further clinical evaluations.

The role of *MET* amplification as a mechanism of primary resistance to cetuximab and panitumumab in mCRC patients has been recently elucidated by Bardelli and colleagues [104]. *MET* amplification was retrospectively found in around 1 % of mCRC samples, in line with previous reports [106]. However, this frequency increased to 12.5 % in a subpopulation of cetuximab-resistant xenopatients bearing wild-type forms of *KRAS*, *NRAS*, *BRAF*, *PIK3CA*, and *HER2*. Notably, only focal, high-grade amplification of the *MET* locus associated with lack of response; conversely, cetuximab proved to be active in tumors with modest gene copy number gains or polysomy of chromosome 7, where the *MET* gene lies [106]. This suggests that resistance is driven by a dosage effect. Multi-arm preclinical trials in MET-positive CRC cell lines and patient-derived xenografts revealed that long-lasting abolition of tumor growth could be achieved through MET inhibition, with or without concurrent interception of EGFR [104, 107]. In coherence, a phase II clinical trial with the primary objective to assess the antitumor efficacy of the dual MET-ALK inhibitor crizotinib in patients with solid tumors (including CRCs) harboring *MET* alterations is currently recruiting participants (NCT02034981, http://clinicaltrials.gov/ct2/results?term=NCT02034981&Search=Search).

*MET* amplification was also found in three out of seven patients who developed acquired resistance, showing mutual exclusivity with secondary *KRAS* mutations. Of note, the *MET* amplicon was detected in circulating, cell-free DNA as early as 3 months after initiation of therapy, before relapse was clinically evident. Like *HER2* amplification and *KRAS* mutations, rare *MET*-amplified cells were found in pretreatment tumor material from one out of three patients with MET-driven acquired resistance, suggesting that preexisting clones were selected under the pressure of anti-EGFR therapy.

### EGFR

Additional genetic alterations within the target oncogene itself, which prevent drug binding and lead to kinase activation even in the presence of the inhibitor, are a common mechanism of both primary and acquired resistance in cancer; a paradigmatic example is provided by the T790M “gatekeeper” secondary mutation in the *EGFR* gene, which installs resistance to reversible EGFR inhibitors in *EGFR*-mutant NSCLC [108]. In colorectal cancer patients, a mutation in the extracellular domain of EGFR (S492R), which abrogates cetuximab binding but retains panitumumab sensitivity, has been recently described as a mechanism of acquired resistance [109, 110] (Fig. 2c). Two mutations in the EGFR kinase domain (codons 714 and 794), which were not detected before EGFR blockade, were identified as circulating mutations by cell-free DNA analysis. Although the functional relevance of these alterations in affecting sensitivity to anti-EGFR moAbs remains to be determined, it is conceivable that they contribute to the onset of secondary resistance [41].

## Other potential biomarkers of drug sensitivity and resistance

The step forward into refining mCRC patient stratification presumably will be the validation of new candidate positive and negative predictors of response to EGFR moAbs. Increased *EGFR* gene copy number could predict response among *KRAS* wild-type patients [53, 91, 111-113], but *EGFR* FISH in mCRCs still needs interlaboratory standardization [75, 114, 115].

Different EGFR-specific ligands could differently influence the clinical activity of cetuximab: while high mRNA levels of either amphiregulin or epiregulin may predict a better response [21, 116-119], high levels of TGF-α as well as HB-EGF could confer lack of sensitivity [107, 118]. These findings, together with the role of other growth factors mentioned in this review, i.e., HGF, sustain the potential but understudied contribution of tumor-stromal interactions in influencing drug response in mCRCs [104, 120, 121].

Controversial data have been reported regarding the predictive role of a number of prognostic biomarkers: for example, IGF-1R overexpression seems to be a favorable prognostic factor [79, 106, 122] while high levels of EphA2, a pro-angiogenic RTK [123, 124], have been associated with poor outcome in mCRC patients treated with cetuximab-based therapy [125]. Biologically, altered tumor angiogenesis as a way to escape cetuximab antitumor activity has been previously reported in CRC cellular models and ascribed to either VEGF protein overexpression or increased VEGFR-1 and VEGFR-2 activation [126, 127]; taken together, these findings suggest that increased expression of pro-angiogenic ligands and cognate receptors (including VEGFs, VEGFRs, and Eph receptors) may dictate sensitivity to anti-EGFR therapy in colorectal tumors. Other “pathway-independent” mechanisms could also have a role in modifying drug response, for example, deregulation of EGFR ubiquitination which affects receptor recycling and expression at the cell membrane [128] (Fig. 2d).

## Ongoing research and challenges

New therapeutic opportunities are currently being offered by genome-scale analyses of CRCs: recurrent mutations, rearrangements, and copy-number alterations have been proposed as therapeutically actionable drivers of colorectal tumorigenesis [129, 130] and will receive further biological validation by future integrated proteogenomics [131]. Promising hints are also emerging from treatments aimed at disrupting immune evasion strategies. As a means to instigate immune suppression, tumor cells often engage immune checkpoint molecules, such as CTLA-4 and PD1, which downregulate pathways of T cell activation. Antibodies against CTLA-4 (ipilimumab) or PD1 (nivolumab) have been shown to induce quick and intense tumor regression in melanoma and NSCLC patients and are currently under clinical investigation in other solid tumors, including CRC [132, 133] (NCT01975831, http://clinicaltrials.gov/ct2/show/NCT01975831).

Although an ever-increasing number of primary and acquired resistance mechanisms have been described until now, mutant *KRAS* is the only validated biomarker in routine practice for selection of mCRC patients to be treated with EGFR-targeted therapies. Thereby, there is a need to develop new models for clinical trials in order to facilitate and accelerate the introduction of other potentially useful biomarkers into clinical practice. Translational research in this context has an unquestionable role. Despite the lack of defined successful endpoints for preclinical models [134], arrest of cell growth and induction of apoptosis in vitro, and especially tumor regression in vivo (ideally in patient-derived xenografts), could have great impact to help design new cancer drug trials. Basket trials, in which patients are treated with different regimens based on their specific genetic profiles, may also optimize outcomes.

Further trial shaping could be provided by genomic analysis from serial biopsies to monitor response evolution and acquisition of genetic or adaptive resistance. However, tumor biopsy may not be representative of the intratumoral and intermetastatic heterogeneity and posttreatment tumor tissue is almost invariably unavailable. Such limitations could be overcome by less-invasive analysis of circulating tumor DNA, which can offer a high degree of sensitivity and specificity to monitor the emergence of drug resistance during the course of treatment [41, 135]. The mechanisms by which ctDNA is released into the circulation and whether multiple metastases shed ctDNA homogeneously are still unclear; however, the proof of principle that such an approach could complement tumor biopsy and provide an early warning of acquired resistance has been established [38, 41, 136].

One way for cancer to escape therapy is by continuous adaptation to the selective pressure of the drug, mainly through tumor genetic heterogeneity and biochemical or transcriptional activation of compensatory feedback loops [137]; exploiting these observations to create a “balance” between drug activity and graded responsiveness of different clones could be useful to delay the onset of resistance and, ideally, to turn cancer into a chronic disease. In this scenario, the lessons learned from metronomic treatment strategies for BRAF V600E melanomas as well as EGFR-mutant NSCLCs suggest that discontinuous dosing of the drug could be a strategy to prevent or retard acquired resistance [138, 139].

Early detection of disease progression calls for hypothesis-driven approaches to contrast outcompetition by subclones exhibiting resistance-conferring mutations. Nonetheless, resistance is pervasive, and observations so far let us conclude that, in most cases, progression to alternative strategies of drug elusion will inevitably occur [109, 140]. Thereby, there is a need to design adaptive drug combinations to achieve tumor response, reduce chances of relapse, and prolong patient survival. In line with this, bioinformatics and systems biology approaches interrogating the huge amount of patient datasets produced until now may provide models and signatures that are more comprehensive and predictive than the mutation status alone [21, 141, 142]. Rational combination therapies guided by real-time monitoring of tumor evolution along treatment, coupled with integrated omics approaches, will ultimately inform trial design to improve patients care in the coming years.

## Final remarks

**Fig. 3:**
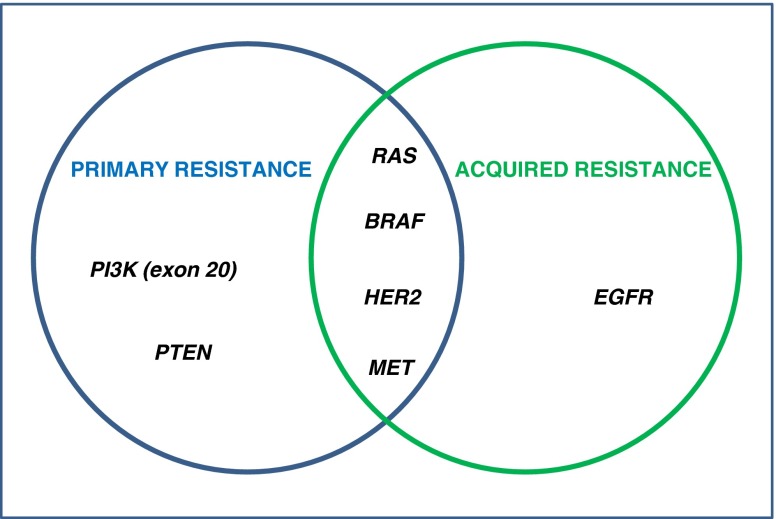
Overlap between molecular biomarkers of primary and acquired resistance in mCRC. Most of the primary and acquired mechanisms of resistance to EGFR-targeted therapies in mCRC overlap. To date, no alterations of the PI3K pathway have been associated with acquired resistance; on the contrary, *EGFR* mutations have never been detected before exposure to EGFR monoclonal antibodies

A decade after the introduction of cetuximab in the treatment of mCRC, much is known about the genetic determinants of primary resistance to anti-EGFR moAbs and initial insights are emerging about the mechanisms underlying acquisition of secondary resistance. The unifying concept is that the very same genetic alterations that account for intrinsic refractoriness also appear to foster progressive lack of response along treatment (Fig. 3), likely due to the presence of preexisting drug-insensitive subclones that are positively selected by continuous EGFR blockade. Future studies are needed to address cogent issues such as modeling tumor heterogeneity along cancer progression and under drug pressure, designing rational combination therapies to target concurrent mutations in the same cells or in different subpopulations, and improving early detection of disease progression.
